# Supplementary material for: Mesenchymal stem cells derived from patients with premature aging syndromes display hallmarks of physiological aging
Source: Life Sci Alliance. 2022 Sep 14;5(12):e202201501. doi: 10.26508/lsa.202201501 (PMC9475049; doi:10.26508/lsa.202201501)
Supplement: Supplementary file 1 [file LSA-2022-01501_TableS1.docx]

Supplementary Table 1. Pathologic cell lines with associated clinics. (AMD: acro-mandibular dysplasia)

| Cell Line | Pathology | Mutation | Clinics |
| --- | --- | --- | --- |
| 1972 | HGPS 1 | LMNA c.1824C>T (p.G608G D50) | Early and accelerated aging  Lipodystrophy  Osteoporosis  Cardiovascular dysfunction |
| 8243 | HGPS 2 |  |  |
| 5968 | HGPS 3 |  |  |
| PC054 | HGPS-L | LMNA c.1868C>G (p.T623S D35) |  |
| OM2 | APS 1 | LMNA c.1583C>T (p.T528M) – Homozygous | Progeria-like, MAD-like and myopathy |
| 13621 | APS 2 | LMNA c.1762T>C (p.C588R) –Heterozygous | AMD_like, no heart attack |
| 10770 | APS 3 | LMNA c.1583C>T and c.1619T>C (p.T528M and p.M540T) – Compound heterozygous | Progeria-like, AMD like and early hypertension |
