# Supplementary material for: Mesenchymal stem cells derived from patients with premature aging syndromes display hallmarks of physiological aging
Source: Life Sci Alliance. 2022 Sep 14;5(12):e202201501. doi: 10.26508/lsa.202201501 (PMC9475049; doi:10.26508/lsa.202201501)
Supplement: Supplementary file 2 [file LSA-2022-01501_TableS2.docx]

Supplementary Table 2. Distribution of differentially methylated probes (DMP) in patient’s cells (MSCs) compared to controls.

|  | CT-Y | | | CT-A | | |
| --- | --- | --- | --- | --- | --- | --- |
|  | APS | HGPS | HGPS-L | APS | HGPS | HGPS-L |
| HyperM | 46192  65.52% | 90051  83.23% | 108982  79.03% | 38883  39.08% | 47473  59.15% | 67476  61.43% |
| HypoM | 24305  34.48% | 18142  16.77% | 28918  20.97% | 60624  60.92% | 32792  40.85% | 42364  38.57% |
