# Supplementary material for: Mesenchymal stem cells derived from patients with premature aging syndromes display hallmarks of physiological aging
Source: Life Sci Alliance. 2022 Sep 14;5(12):e202201501. doi: 10.26508/lsa.202201501 (PMC9475049; doi:10.26508/lsa.202201501)
Supplement: Supplementary file 3 [file LSA-2022-01501_TableS3.docx]

Supplementary Table 3. Distribution of Hypermethylated (top) and Hypomethylated (bottom) probes relative to CpGs, CpG islands, shores (2kb flanking CpG islands), shelves (2kb extending from shores) or openseas (isolated CpG in the rest of the genome) in patient cells (MSCs) compared to controls.

| Hypermethylated probes | | | | | | | |
| --- | --- | --- | --- | --- | --- | --- | --- |
|  | CT-Y | | | CT-A | | |  |
|  | APS | HGPS | HGPS-L | APS | HGPS | HGPS-L | 850K |
| Islands | 2415  5.23% | 5977  6.64% | 6629  6.08% | 2708  6.96% | 2860  6.02% | 3370  4.99% | 161441  18.63% |
| Open sea | 33959  73.52% | 68373  75.93% | 84407  77.45% | 25959  66.76% | 34235  72.11% | 51399  76.17% | 488876  56.42% |
| Shelf | 2777  6.01% | 5074  5.63% | 6265  5.75% | 2472  6.36% | 2817  5.93% | 3964  5.87% | 61691  7.12% |
| Shore | 7041  15.24% | 10627  11.8% | 11681  10.72% | 7744  19.92% | 7561  15.93% | 8743  12.96% | 154546  17.83% |
|  | Hypomethylated probes | | | | | |  |
|  | CT-Y | | | CT-A | | |  |
|  | APS | HGPS | HGPS-L | APS | HGPS | HGPS-L | 850K |
| Islands | 3169  13.04% | 2461  13.57% | 3756  12.99% | 7217  11.9% | 3354  10.23% | 4310  10.17% | 161441  18.63% |
| Open sea | 13628  56.07% | 9508  52.41% | 15714  54.34% | 38134  62.9% | 19559  59.65% | 24953  58.9% | 488876  56.42% |
| Shelf | 1312  5.4% | 1062  5.85% | 1596  5.52% | 3583  5.91% | 2065  6.3% | 2594  6.12% | 61691  7.12% |
| Shore | 6196  25.49% | 5111  28.17% | 7852  27.15% | 11690  19.28% | 7814  23.83% | 10507  24.8% | 154546  17.83% |
