# Supplementary material for: Mesenchymal stem cells derived from patients with premature aging syndromes display hallmarks of physiological aging
Source: Life Sci Alliance. 2022 Sep 14;5(12):e202201501. doi: 10.26508/lsa.202201501 (PMC9475049; doi:10.26508/lsa.202201501)
Supplement: Supplementary file 4 [file LSA-2022-01501_TableS4.docx]

Supplementary Table 4. Distribution of Hypermethylated (top) and hypomethylated (bottom) probes corresponding to gene functional elements. E.g : first exon, 3’ UTR, 5’UTR, gene bodies, Exon boundaries, Internal genomic regions (IGR), 1500 bp from transcription start sites (TSS1500) or 2000 bp from transcription start sites (TSS200).

| Hypermethylated probes | | | | | | | |
| --- | --- | --- | --- | --- | --- | --- | --- |
|  | CT-Y | | | CT-A | | |  |
|  | APS | HGPS | HGPS-L | APS | HGPS | HGPS-L | 850K |
| 1st exon | 665  1.44% | 1633  1.81% | 1819  1.67% | 589  1.51% | 726  1.53% | 910  1.35% | 26419  3.05% |
| 3’UTR | 894  1.94% | 1711  1.9% | 2059  1.89% | 803  2.07% | 953  2.01% | 1300  1.93% | 21557  2.49% |
| 5’ UTR | 3125  6.77% | 6163  6.84% | 8150  7.48% | 3175  8.17% | 3568  7.52% | 5350  7.93% | 72994  8.42% |
| Body | 16358  35.41% | 30664  34.05% | 39417  36.17% | 14631  37.63% | 17599  37.07% | 26229  38.87% | 317777  36.67% |
| Exon Bnd | 217  0.47% | 455  0.51% | 536  0.49% | 208  0.53% | 255  0.54% | 334  0.49% | 5678  0.66% |
| IGR | 18.09  39.64% | 36668  40.72% | 43398  39.82% | 13349  34.33% | 17668  37.22% | 25578  37.91% | 249956  28.84% |
| TSS1500 | 5043  10.92% | 8972  9.96% | 9434  8.66% | 4651  11.96% | 5015  10.56% | 5695  8.44% | 107064  12.36% |
| TSS200 | 1581  3.42% | 3785  4.2% | 4169  3.83% | 1477  3.8% | 1689  3.56% | 2080  3.08% | 65109  7.51% |
|  | Hypomethylated probes | | | | | |  |
|  | CT-Y | | | CT-A | | |  |
|  | APS | HGPS | HGPS-L | APS | HGPS | HGPS-L | 850K |
| 1st exon | 523  2.15% | 427  2.35% | 575  1.99% | 1408  2.32% | 712  2.17% | 828  1.95% | 26419  3.05% |
| 3’UTR | 519  2.15% | 354  1.95% | 668  2.31% | 1155  1.91% | 572  1.74% | 890  2.1% | 21557  2.49% |
| 5’ UTR | 1986  8.17% | 1601  8.82% | 2332  8.06% | 4967  8.19% | 2743  8.36% | 3193  7.54% | 72994  8.42% |
| Body | 8547  35.17% | 6342  34.96% | 9983  34.52% | 20555  33.91% | 11307  34.48% | 14349  33.87% | 317777  36.67% |
| Exon Bnd | 102  0.42% | 75  0.41% | 170  0.59% | 265  0.44% | 127  0.39% | 189  0.45% | 5678  0.66% |
| IGR | 7782  32.02% | 5550  30.59% | 8894  30.76% | 21487  35.44% | 11297  34.45% | 14529  34.3% | 249956  28.84% |
| TSS1500 | 3601  14.82% | 2826  15.58% | 4800  16.6% | 7298  12.04% | 4364  13.31% | 6283  14.83% | 107064  12.36% |
| TSS200 | 1245  5.12% | 967  5.33% | 1496  5.17% | 3489  5.76% | 1670  5.09% | 2103  4.96% | 65109  7.51% |
