# Supplementary material for: Mesenchymal stem cells derived from patients with premature aging syndromes display hallmarks of physiological aging
Source: Life Sci Alliance. 2022 Sep 14;5(12):e202201501. doi: 10.26508/lsa.202201501 (PMC9475049; doi:10.26508/lsa.202201501)
Supplement: Supplementary file 5 [file LSA-2022-01501_TableS5.docx]

Supplementary Table 5. Association to functional chromatin elements using ChromHMM track (MSCs E025 Cells, Roadmap Epigenomics Mapping Consortium)

|  | Hypermethylated probes | | | | | |  |
| --- | --- | --- | --- | --- | --- | --- | --- |
|  | CT-Y | | | CT-A | | |  |
|  | APS | HGPS | HGPS-L | APS | HGPS | HGPS-L | 850K |
| TSSActive | 5833  12.63% | 12269  13.62% | 14703  13.49% | 6298  16.2% | 7542  15.89% | 9756  14.46% | 202331  23.49% |
| Transcription | 5006  10.84% | 7016  7.79% | 9465  8.68% | 6188  15.91% | 6025  12.69% | 7923  11.74% | 174982  20.31% |
| Enhancer | 7650  16.56% | 14999  16.66% | 24461  22.44% | 7834  20.15% | 10643  22.42% | 19471  28.86% | 105218  12.22% |
| Heterochromatin | 626  1.36% | 1418  1.57% | 1406  1.29% | 282  0.73% | 480  1.01% | 645  0.96% | 8497  0.99% |
| TSSBivalent | 676  1.46% | 1203  1.34% | 1273  1.17% | 656  1.69% | 671  1.41% | 757  1.12% | 22519  2.61% |
| Enhancerbivalent | 413  0.89% | 651  0.72% | 788  0.72% | 381  0.98% | 419  0.88% | 554  0.82% | 6185  0.72% |
| RepressivePolycomb | 12088  26.17% | 25685  28.25% | 28161  25.84% | 9999  25.72% | 11693  24.63% | 14528  21.53% | 184472  21.42% |
| Quiescent | 13900  30.09% | 26810  29.77% | 28725  26.36% | 7245  18.63% | 10000  21.06% | 13842  20.51% | 157159  18.25% |
|  | Hypomethylated probes | | | | | |  |
|  | CT-Y | | | CT-A | | |  |
|  | APS | HGPS | HGPS-L | APS | HGPS | HGPS-L | 850K |
| TSSActive | 6178  25.42% | 4318  23.8% | 7274  25.15% | 13559  22.37% | 6821  20.8% | 9121  21.53% | 202331  23.49% |
| Transcription | 3107  12.78% | 2888  15.92% | 4802  16.61% | 6292  10.38% | 4317  13.16% | 5883  13.89% | 174982  20.31% |
| Enhancer | 3892  16.01% | 2722  15% | 4013  13.88% | 10701  17.65% | 5610  17.11% | 6109  14.42% | 105218  12.22% |
| Heterochromatin | 158  0.65% | 111  0.61% | 204  0.71% | 311  0.51% | 132  0.4% | 319  0.75% | 8497  0.99% |
| TSSBivalent | 877  3.61% | 684  3.77% | 944  3.26% | 1765  2.91% | 1092  3.33% | 1326  3.13% | 22519  2.61% |
| Enhancerbivalent | 347  1.43% | 228  1.26% | 329  1.14% | 773  1.28% | 438  1.34% | 562  1.33% | 6185  0.72% |
| RepressivePolycomb | 6161  25.35% | 4816  26.55% | 6914  23.91% | 16561  27.32% | 9321  28.42% | 11571  27.31% | 184472  21.42% |
| Quiescent | 3585  14.75% | 2375  13.09% | 4438  15.35% | 10662  17.59% | 5061  15.43% | 7473  17.64% | 157159  18.25% |
