# Supplementary material for: Mesenchymal stem cells derived from patients with premature aging syndromes display hallmarks of physiological aging
Source: Life Sci Alliance. 2022 Sep 14;5(12):e202201501. doi: 10.26508/lsa.202201501 (PMC9475049; doi:10.26508/lsa.202201501)
Supplement: Supplementary file 6 [file LSA-2022-01501_TableS6.docx]

Supplementary Table 6. Overlap between Downregulated DEGs and aggregated Hypermethylated DMPs; Upregulated DEGs and aggregated Hypomethylated DMPs, respectively. Percentage of overlap are given considering the overall DEGs and Aggregated DMPs.

|  | Hypermethylated | | | | | |
| --- | --- | --- | --- | --- | --- | --- |
|  | CT-Y | | | CT-A | | |
|  | APS | HGPS | HGPS-L | APS | HGPS | HGPS-L |
| Downregulated DEGs | 181 | 210 | 618 | 218 | 358 | 1075 |
| Aggregated DMPs | 8258 | 12381 | 12856 | 6446 | 9425 | 10400 |
| Overlap | 79  43.65% | 71  33.81% | 279  45.15% | 44  20.18% | 120  33.52% | 402  37.40% |

|  | Hypomethylated | | | | | |
| --- | --- | --- | --- | --- | --- | --- |
|  | CT-Y | | | CT-A | | |
|  | APS | HGPS | HGPS-L | APS | HGPS | HGPS-L |
| Upregulated DEGs | 116 | 242 | 574 | 106 | 229 | 924 |
| Aggregated DMPs | 5643 | 3094 | 4044 | 9463 | 5464 | 6131 |
| Overlap | 39  33.62% | 39  16.12% | 85  14.81% | 21  19.81% | 50  21.83% | 188  20.35% |
