# Supplementary material for: Mesenchymal stem cells derived from patients with premature aging syndromes display hallmarks of physiological aging
Source: Life Sci Alliance. 2022 Sep 14;5(12):e202201501. doi: 10.26508/lsa.202201501 (PMC9475049; doi:10.26508/lsa.202201501)
Supplement: Supplementary file 7 [file LSA-2022-01501_TableS7.docx]

Supplementary Table 7. List of RT-qPCR primers

| Gene | Forward | Reverse |
| --- | --- | --- |
| HPRT | TGATAGATCCATTCCTATGACTGTAGA | CAAGACATTCTTTCCATGTAAAGT |
| PPIA | ATGCTGGACCCAACACAAAT | TCTTTTATCTTGCCAAACACC |
| FOXC1 | TGAACGGCAATAGTAGCTGTCA | GGACGTGCGGTACAGAGAC |
| FOXC2 | GGGGACCTGAACCACCTC | AACATCTCCCGCACGTTG |
| COL1A1 | CCCAAGGCTTCCAAGGTC | GGACGACCAGGTTTTCCAG |
| COL1A2 | TGGTGAAGTGGGTCTTCCA | ACCCTTGGCACCAGTAAGG |
| GTF2H2 | CATGCCTGGACATACAAGTCGAG | CAGTGCAAACGCGAACTTCTGC |
| TGFB2 | CAAAGGGTACAATGCCAACTT | GCAGATGCTTCTGGATTTATGG |
| FGFR2 | CCAAAATGGGAGTTTCCAAG | CATGACCACTTGCCCAAAG |
| TBX20 | AGCAAATGGCCAAAATTGC | GATGGTTGGAAACATCCTCCT |
